# CD68, CD163, and matrix metalloproteinase 9 (MMP-9) co-localization in breast tumor microenvironment predicts survival differently in ER-positive and -negative cancers

**DOI:** 10.1186/s13058-018-1076-x

**Published:** 2018-12-17

**Authors:** Vasiliki Pelekanou, Franz Villarroel-Espindola, Kurt A. Schalper, Lajos Pusztai, David L. Rimm

**Affiliations:** 1 Department of Pathology, Yale School of Medicine, 310 Cedar Street, P.O. Box 208023, New Haven, CT 06520 USA; 2 Department of Medical Oncology, Yale School of Medicine, 330 Cedar Street, New Haven, 06520 CT USA; 3 Sanofi US Services Inc., Bridgewater Township, USA

**Keywords:** Tumor-associated macrophages, Matrix metalloproteinase-9, Breast cancer

## Abstract

**Background:**

The role of tumor-associated macrophages (TAMs) in the cancer immune landscape and their potential as treatment targets or modulators of response to treatment are gaining increasing interest. TAMs display high molecular and functional complexity. Therefore their objective assessment as breast cancer biomarkers is critical. The aims of this study were to objectively determine the *in situ* expression and significance of TAM biomarkers (CD68, CD163, and MMP-9) in breast cancer and to identify subclasses of patients who could benefit from TAM-targeting therapies.

**Methods:**

We measured CD68, CD163, and MMP-9 protein expression in formalin-fixed paraffin-embedded tissues of breast carcinomas represented in tissue microarray format using multiplexed quantitative immunofluorescence (QIF) in two independent Yale cohorts: cohort A—*n* = 398, estrogen receptor–positive (ER^+^) and ER^−^ cases—and the triple-negative breast cancer (TNBC)-only cohort B (*n* = 160). Associations between macrophage markers, ER status, and survival were assessed. Protein expression measured by QIF was compared with mRNA expression data from the METABRIC study.

**Results:**

All three macrophage markers were co-expressed, displaying higher expression in ER^−^ cancers. High pan-macrophage marker CD68 correlated with poorer overall survival (OS) only in ER^−^ cases of cohort A (*P* = 0.02). High expression of CD163 protein in TAMs was associated with improved OS in ER^−^ cases (cohort A, *P* = 0.03 and TNBC cohort B, *P* = 0.04, respectively) but not in ER^+^ cancers. MMP-9 protein was not individually associated with OS. High expression of MMP-9 in the CD68^+^/CD163^+^ TAMs was associated with worse OS in ER^+^ tumors (*P* <0.001) but not in ER^−^ cancers. In the METABRIC dataset, mRNA levels followed the co-expression pattern observed in QIF but did not always show the same trend regarding OS.

**Conclusions:**

Macrophage activity markers correlate with survival differently in ER^+^ and ER^−^ cancers. The association between high co-expression and co-localization of MMP-9/CD163/CD68 and poor survival in ER^+^ cancers suggests that these cancers may be candidates for macrophage-targeted therapies.

**Electronic supplementary material:**

The online version of this article (10.1186/s13058-018-1076-x) contains supplementary material, which is available to authorized users.

## Background

The recent success of immunotherapies has increased interest in the immune status of breast cancer [1, 2]. Tumor-infiltrating lymphocytes (TILs) represent a mechanism for assessment of immune status. Studies have shown that TILs are prognostic, particularly for estrogen receptor–negative (ER^−^) and highly proliferative ER^+^ cancers [3–6]. Despite their prognostic value, high TILs counts are found in only a small subset of breast carcinomas [6–9] whereas macrophages are the most common immune cells [10]. Tumor-associated macrophages (TAMs) are pleiotropic regulators of tumor cells and microenvironment, modulating tumor growth, activation, and response to therapy [11–16]. Novel immunomodulatory agents specifically targeting TAM proteins, such as colony-stimulating factor 1 receptor (CSF-1R) and matrix metalloproteinase-9 (MMP-9), are currently in the pipeline and/or under clinical testing as mono-therapy or in combination with conventional established therapies and/or immune checkpoint inhibitors. However, TAM biomarkers’ potential in companion diagnostics remains unclear. Unlike TILs, TAMs cannot be assessed by standardized methods on hematoxylin/eosin (H&E) slides. Although they can be seen, they are largely ignored with respect to morphologic diagnostics. Similarly, their molecular assessment has been shown to be highly variable and highly heterogeneous, resulting in the lack of adequate cell models and discrepancies between murine models and human macrophage biologic features [17].

Conventionally, TAMs have been divided into M1 and M2 subtypes to define their polarization status. In general, M1-polarized macrophages mediate resistance to intracellular pathogens and tumors (Th1-driven responses) whereas M2-polarized macrophages mediate resistance to parasites, immunoregulation, tissue repair, and immuno-tolerance against tumors. However, this conventional M1/M2 dichotomy is controversial and not consistently representative of the TAM functional continuum [14]. Previous reports have associated TAMs with outcome of breast cancer patients but with contradictory results [18–23]. In most cases, their prognostic assessment is limited by *in situ* single-marker, semi-quantitative chromogenic detection of “traditional” biomarkers and their M1/M2-like features (for example, CD68, CD163, metalloproteinases, and arginase) or within high-throughput genomic data, lacking key spatial context, neither of which has seen adoption in the clinical setting.

Within proteins that are differentially expressed in M1- and M2-like TAM subtypes, we were particularly interested in MMP-9, a member of MMP family, because it has been shown to play a role in extracellular matrix remodeling and invasion in breast cancer. Specific MMP-9 inhibitors, such as GS-5745 (Andecaliximab) [24, 25], are being tested in clinical trials in combination with chemotherapy or immune checkpoint inhibitors in order to block paracrine signaling and metastasis and to alter the immune microenvironment within the tumor. In preclinical models, inhibition of MMP-9 has been shown to inhibit immune-suppressive myeloid cell polarization, regulatory T cells, desmoplastic reaction, and effector T-cell trafficking. These data suggest that MMP-9 inhibition could modulate immune suppression. In breast cancer, however, MMP-9 has been traditionally studied as a tumor cell–derived peptidase and, to a lesser extent, as an immune cell protein, participating in regulation of tumor microenvironment and immune cell infiltrate.

Here, we have objectively and simultaneously measured *in situ* the expression of TAM biomarkers (CD68, CD163, and the druggable target MMP-9) in two distinct breast cancer cohorts by using the validated quantitative immunofluorescence (QIF) AQUA method [26]. We compared our results with mRNA expression data from the largest available breast cancer series (METABRIC) to evaluate whether protein expression combined with spatial distribution would be more informative as biomarker. Our objectives were to subclassify TAMs as the most prevalent breast cancer immune cells and to determine how their polarization is associated with breast tumors’ molecular phenotype and patients’ outcome and how these features could be further exploited in pharmacologic modulation of macrophage function.

## Methods

### Patient cohorts and tissue microarrays

Samples from two retrospective collections of breast cancer from Yale University (cohorts A and B) treated surgically primarily were used. The major clinicopathological characteristics and available treatment information of the cohorts are presented in Table 1. The cohorts consist of retrospective stage I–III breast cancer collections represented in a tissue microarray (TMA) format: cohort A (*n* = 398, comprising both ER^+^ and ER^−^ cases collected between 1976 and 2005) and cohort B, comprising exclusively triple-negative breast cancer (TNBC) (*n* = 160, collected between 1998 and 2004 and treated with standard chemotherapy). ER, PR, and HER2 status was determined by the local institution’s clinical laboratory.

**Table 1:** Clinicopathological characteristics of cohorts A and triple-negative breast cancer cohort B

Parameter	Cohort A	Cohort B (TNBC)
	*N* (%)	*N* (%)
All patients	398	156
Age, years		
<50	124 (31.2)	72 (46.15)
≥50	257 (64.6)	80 (50.1)
Unknown	17 (4.3)	4 (2.56)
Nodal status		
Positive	78 (19.6)	15 (9.6)
Negative	203 (51.0)	39 (25)
Unknown	117 (29.4)	104 (66.6)
Tumor size		
<2 cm	217 (54.5)	44 (28.2)
2–5 cm	73 (17.3)	74 (47.43)
Unknown	108 (27)	38 (24.35)
Grade		
1–2	97 (24.37)	45 (28.84)
3	103 (25.87)	78 (50.00)
NA/Unknown	198 (49.7)	32 (2)
ERα		
Positive (1–3)	264 (66.3)	
Negative (0)	89 (22.3)	
Unknown	45 (11.3)	
HER2		
Positive (3+)	20 (5)	
Negative (0–1+)	272 (68.3)	
Unknown/Equivocal	106 (26.6)	
Adjuvant treatment		
Hormonal only	93 (23.4)	
Chemotherapy only	72 (18.1)	
Hormonal+Chemo	48 (12.1)	
None	89 (22.4)	
Unknown	96 (24.1)	
Follow-up (m)		
Median (range)	139 (3–385)	52 (4–231)

*Abbreviations*: *ERα* estrogen receptor alpha, *NA* not available, *TNBC* triple-negative breast cancer

TMAs were prepared using 0.6-mm tissue cores, each in twofold redundancy using standard procedures. The TMAs were constructed by selecting areas of donor blocks containing viable tumor cells and stromal elements (as assessed by an expert pathologist using H&E stain) and without enriching for specific tumor regions (for example, tumor margin versus tumor core). All tissue was used after patient consent and approval from Yale Human Investigation Committee protocol #9505008219 for cases from Yale University, which approved the patient consent forms or, in some cases, waiver of consent (since these were otherwise discarded tissues collected during routine medical care).

### Quantitative immunofluorescence

Multiplexed QIF staining for TAM protein detection of CD68, CD163, MMP-9, cytokeratin, and 4′,6-diamidino-2-phenylindole (DAPI) was simultaneously quantified on the same slide for every patient. Briefly, fresh cuts of TMAs were deparaffinized and rehydrated before undergoing antigen retrieval using an EDTA buffer (pH = 8) for 20 min at 97 °C (PT module, Lab Vision, Thermo Fisher Scientific, Waltham, MA, USA). Slides were then incubated with dual endogenous peroxidase block (Dako, Glostrup, Denmark) for 10 min to block endogenous peroxidase activity and incubated with 0.3% bovine serum albumin in a 0.05% Tween solution for 30 min to block non-specific antigens. Fluorescent staining for pancytokeratin, CD68, CD163, and MMP-9 was performed by using a sequential multiplexed protocol with different isotype-specific primary antibodies. Antibodies against these targets were used to detect epithelial tumor cells (cytokeratin 8 and 18, clone M3515, Abcam, Cambridge, UK), all macrophages (CD68, mouse monoclonal IgG3, clone PG-M1, Dako, Glostrup, Denmark 0.3 μg/mL), M2-like macrophages (CD163, mouse monoclonal IgG1, clone CD163-L-U (Leica, Novocastra, Wetzlar, Germany, 0.006 μg/mL), and MMP-9 (rabbit monoclonal, clone DX6O3H-XP, Cell Signaling Technology, Danvers, MA, USA, 0.58 μg/mL). All nuclei were then tagged with DAPI (Life Technologies, Carlsbad, CA, USA). Secondary antibodies conjugated to horseradish peroxidases (HRPs) and specific to each primary antibody isotype were used (anti-rabbit EnVision, Dako; anti-mouse IgG1, eBioscience, San Diego, CA, USA; anti-mouse IgG3, ab97260, Abcam), while tyramide-bound fluorophores were added to bind to the HRPs (biotinylated tyramide, PerkinElmer, Waltham, MA, USA; streptavidin-Alexa750, Life Technologies; Cy3 TSA™ Plus fluorescein-tyramide; cyanine 5, both from PerkinElmer, Waltham, MA, USA). A fluorophore-conjugated goat anti-chicken secondary antibody was used against the cytokeratin antibody (goat anti-chicken Alexa488, Life Technologies). Residual, unbound HRPs were blocked between incubations with a 0.15% hydrogen peroxide benzoic hydrazide solution. Sections from a tonsil/lymph node TMA were included as control for macrophage and lymphoid cells. Validation data are provided in Additional file 1: and Additional file 2: Figures S1–S4.

### Fluorescence measurement and scoring

Quantitative measurement of fluorescent signal was obtained by using automated quantitative analysis (AQUA^®^) technology (Navigate, Carlsbad, CA, USA), which allows objective and accurate measurement of protein expression within marker-defined compartments, as previously described [26]. AQUA technology does not require feature-based image fractionation but rather allows detection of biomarker expression within specific subcellular compartments, as defined by antibody-conjugated fluorophore labelling and co-localization of the target of interest with cytoplasmic or nuclear staining. The fluorescent intensity is measured and divided by the compartment area to yield a quantitative, continuous, and reproducible score for each field of view. Five monochromatic images, each corresponding to a different fluorescent channel (DAPI, fluorescein isothiocyanate, Cy3 Plus, Cy5, and Cy7), were captured for each TMA spot by using a PM-2000 image workstation (HistoRx, Branford, CT). In order to accurately quantify the signal intensity of the emission wavelengths in each fluorescent channel with AQUA^®^ software (Navigate, Biopharma), areas lacking invasive breast carcinomas as demonstrated by cytokeratin staining—for example, normal breast tissue, ductal carcinoma *in situ* (DCIS)—were excluded from analysis, as were any experimental artifacts (for example, folded or damaged tissue). QIF scores were generated for each channel. Scores were normalized to exposure time and bit depth during time of capture to allow proper comparison across all samples. Twofold redundancy was applied, and the average QIF scores of a given marker were used.

### RNA data

Publicly available data from the METABRIC study (2509 samples) [27, 28] were downloaded from http://www.cbioportal.org/ (version 1.4.2 snapshot). We have retrieved mRNA data for MMP-9, CD68, and CD163 genes and analyzed their co-expression, co-occurrence, or mutual exclusion as well as their expression correlation with patients’ clinicopathological data (age, ER status, grade, tumor size, and PAM50 subtype).

### Statistical analysis

AQUA scores were used as a continuous variable or dichotomized into high and low marker expression. Our clinical endpoint was overall survival (OS), as complete data on relapse-free survival and adjuvant treatment were available for cohort A only and not cohort B. For every cohort, AQUA QIF scores from two independent cores were averaged and used for final analysis. Positivity was assessed visually by an expert pathologist (VP). Median QIF score value was used as cut point to determine low and high cases. Spearman’s correlation coefficient (R) was used to assess the reproducibility of the assay between consecutive sections of the index array. Differences between QIF signals between groups were analyzed by using Fisher’s exact test, and two-sided *P* values were considered statistically significant if less than 0.05. Linear regression coefficients and Spearman’s correlations were calculated to determine the association between continuous scores. GraphPad Prism 7.01 software (GraphPad Software, La Jolla, CA, USA) was used for Kaplan–Meier OS QIF curves. JMP 11.0 was used for multivariate analysis. X-tile software (Rimm Lab, Yale Scool of Medicine, New Haven, CT, https://medicine.yale.edu/lab/rimm/research/software.aspx) was used for mRNA survival analysis and optimal cut-point determination.

## Results

### The prognostic value of CD68 and CD163 macrophage markers is influenced by ER status of breast carcinomas

**Fig. 1:**
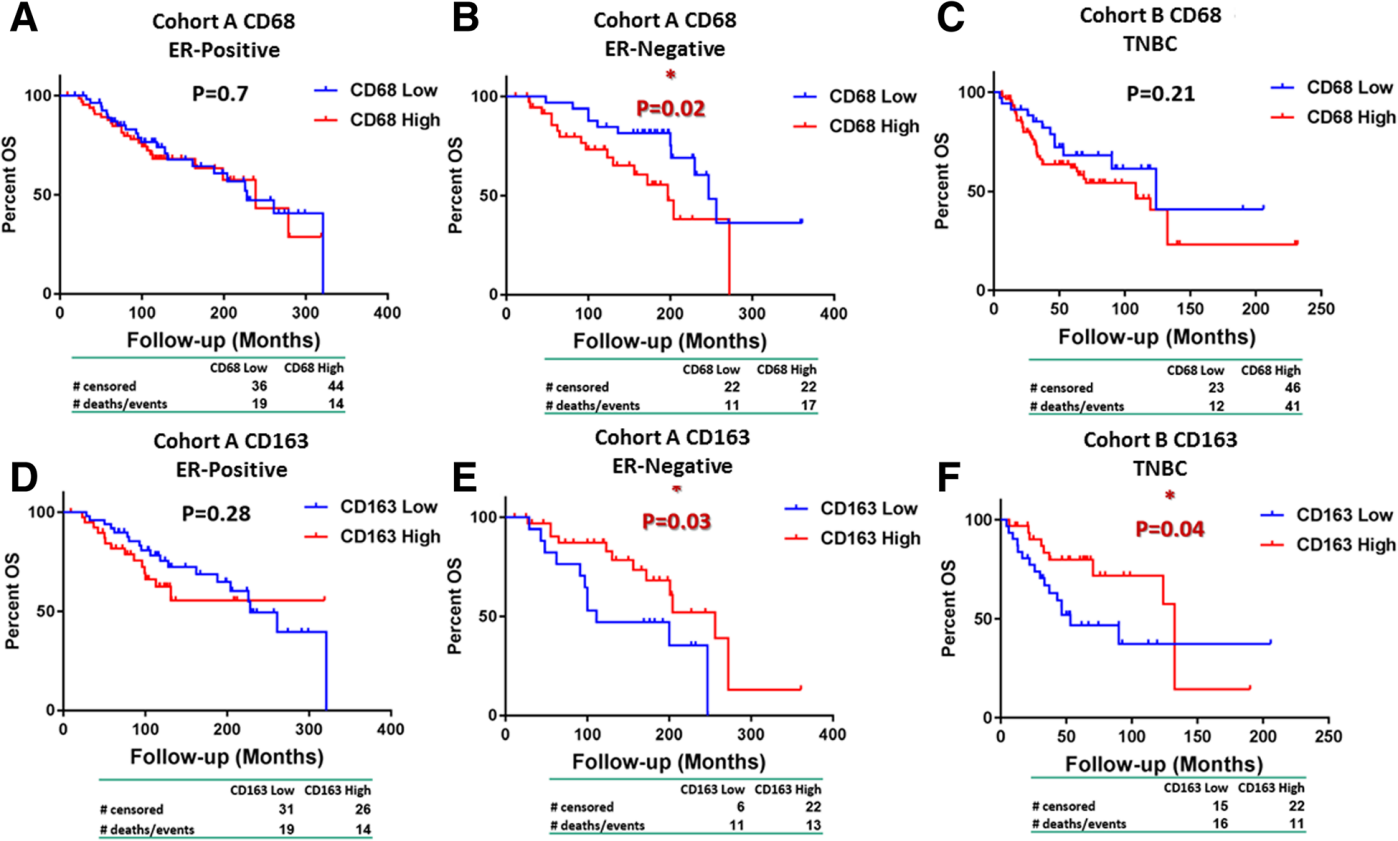
Survival analysis of CD68 and CD163 expression based on estrogen receptor (ER) status. Kaplan–Meier curves of CD68 (upper panel) in ER-positive (**a**) and ER-negative (**b**) cases of cohort A and triple-negative breast cancer (TNBC) cohort B (**c**). Kaplan–Meier curves of CD163 (lower panel) in ER-positive (**d**) and ER-negative (**e**) cases of cohort A and TNBC cohort B (**f**)

ER is amongst the most important biomarkers for breast cancer; thus, all three TAM biomarkers (CD68, CD163, and MMP-9) are examined in the context of ER status. High expression of pan-macrophage marker CD68 alone was associated with worse OS in the subset of ER^−^ tumors in cohort A (*P* = 0.02) but not in TNBC cohort B or the ER^+^ subset of cohort A (Fig. 1a–c). Co-expression of the M2-like biomarker CD163 with CD68 suggests a different subclass of TAMs (CD163^+^/CD68^+^) and inverts the prognostic value. Higher levels of CD163 within CD68^+^TAM infiltrate were associated with improved survival in ER^−^ cases of both cohorts (ER^−^ cases of cohort A, *P* = 0.03 and TNBC cohort B, *P* = 0.04, respectively) (Fig. 1d–f). We did not find any statistically significant association with patient outcome and expression of CD68 alone or co-expression of CD68/CD163 in ER^+^ cases.

### TAMs are the main cell population expressing MMP-9, predominantly in the stroma and perivascular areas

**Fig. 2:**
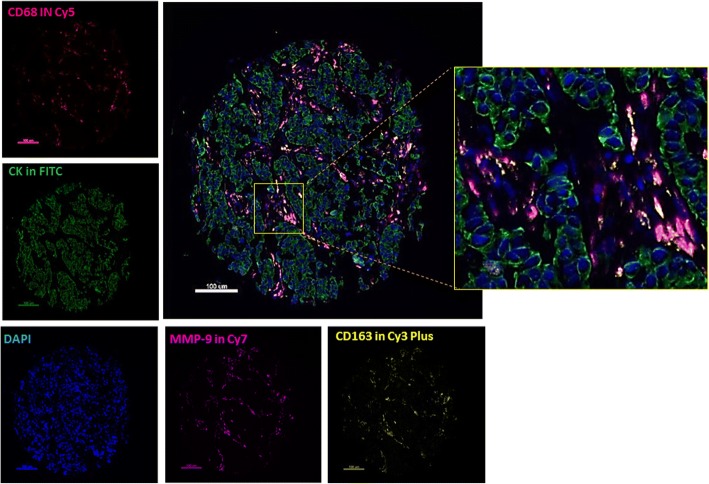
Detection of CD68, CD163, and matrix metalloproteinase 9 (MMP-9) using multiplex quantitative immunofluorescence (QIF) in breast cancer. Representative fluorescence images showing the detection of tumor-associated macrophage (TAM) subsets in breast cancer samples by simultaneous staining of DAPI (blue channel), cytokeratin (fluorescein isothiocyanate, green channel), CD68 (Cy5, red channel), CD163 (Cy3 Plus, yellow channel), and MMP-9 (Cy7, magenta channel). The insert shows higher magnification of stromal TAMs. Bar = 100 μm

TAMs, including all CD68^+^ macrophages, as well as CD68^+^/CD163^+^ cells were found at the stroma and perivascular areas or filling ductal-like structures and inter-epithelial cell gaps within the tumor islets (Fig. 2). The correlation between CD68 and CD163 scores was *r* = 0.6 for both cohorts. MMP-9 expression was restricted to stromal cells and especially TAMs, following their pattern of distribution (Fig. 1 as well as QIF distributions scores). No tumor MMP-9 expression was observed. All cases showed some expression of CD68, CD163, and MMP-9, and there was a wide range of QIF scores (Additional file 2: Figure S5A–D). All three markers were significantly correlated (*P* <0.001).

### TAM biomarkers CD68, CD163, and MMP-9 association with clinicopathological data

**Fig. 3:**
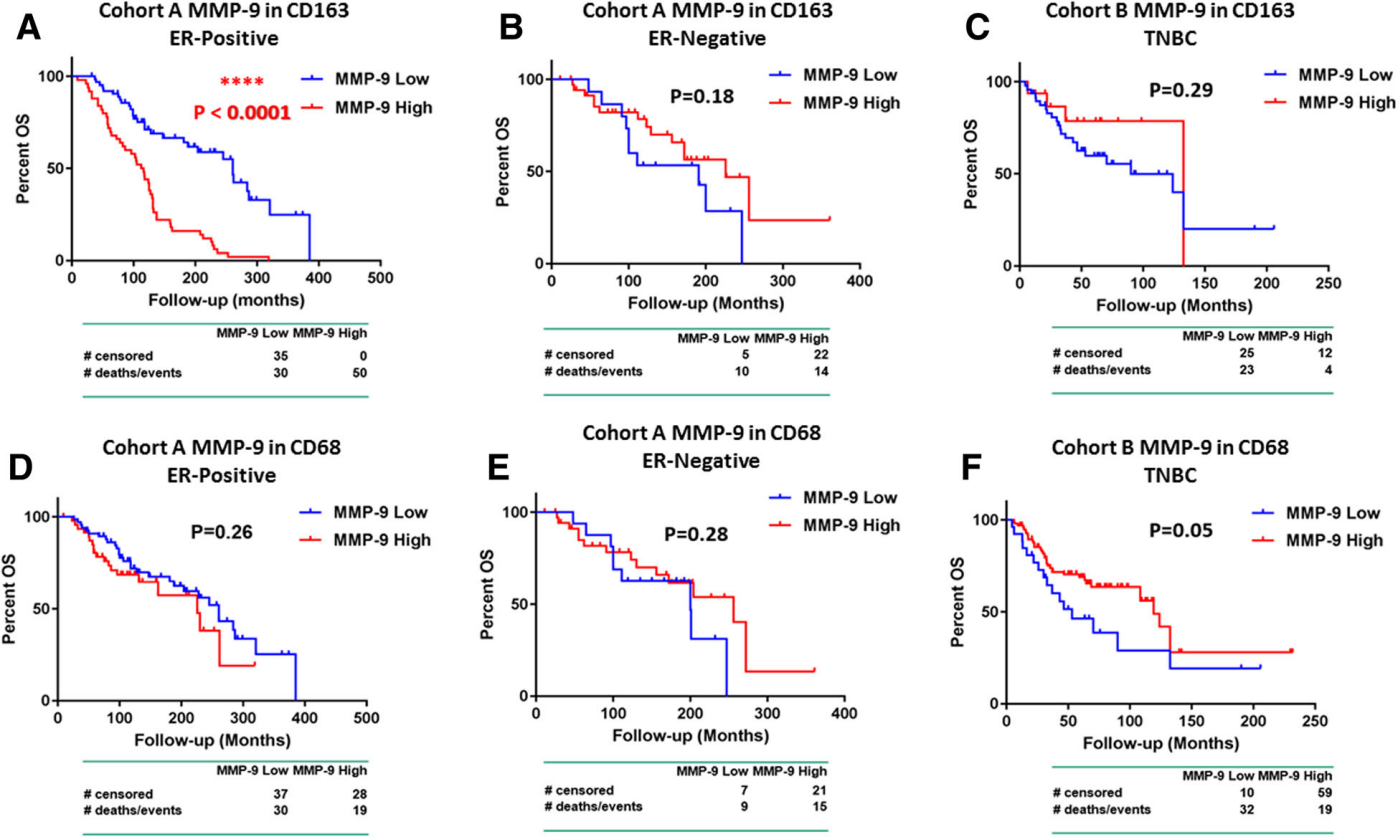
Survival analysis of matrix metalloproteinase 9 (MMP-9) expression in CD68^+^ and CD163^+^ tumor-associated macrophages (TAMs) based on estrogen receptor (ER) status. Kaplan–Meier curves of MMP-9 expression in CD163^+^ TAMs (upper panel) in ER-positive (**a**) and ER-negative (**b**) cases of cohort A and triple-negative breast cancer (TNBC) cohort B (**c**). Kaplan–Meier curves of MMP-9 expression in CD68^+^ TAMs (lower panel) in ER-positive (**d**) and ER-negative (**e**) cases of cohort A and TNBC cohort B (**f**)

High MMP-9 expression had a significant association with impaired OS in ER^+^ tumors of cohort A (Fig. 3a, *P* <0.001) only in CD68^+^/CD163^+^ polarized TAMs but not in ER^−^ (Fig. 3b) or TNBC cases (Fig. 3c) or when measured in all CD68^+^ macrophages without M2 polarization (CD163) taken into account (Fig. 3d, e). In TNBC cohort B, high MMP-9 levels within CD68^+^ macrophages had a non-significant trend for improved OS (Fig. 3f, *P* = 0.05). In multivariate analysis, neither MMP-9 nor CD68 and CD163 were independent prognostic factors in either of the two cohorts (Table 2). We found no prognostic significance when QIF scores of all three TAM biomarkers were analyzed as continuous variables (not shown).

**Table 2:** Overall survival multivariate analysis

	Cohort A			Cohort B		
	HR	CI 95%	*P* value	HR	CI 95%	*P* value
MMP-9 in CD68	1.26	0.20–10.35	0.81			0.2
MMP-9 in CD163	0.46	0.07–2.23	0.35	1.99	0.27–13.44	0.47
CD163	1.71	0.88–3.5	0.11	0.45	0.08–2.25	0.33
CD68	1.58	0.66–4.02	0.3	13.44	0.76–382.29	0.07
Age >50 years	6.24	2.13–20.21	0.0006	2.6	0.27–58.1	0.41
Size >2 cm	1.14	0.49–2.69	0.75	3.4	0.11–103	0.42
Grade	1.67	0.87–3.16	0.11	0.41	0.03–9.36	0.51

*Abbreviations*: *CI* confidence interval, *HR* hazard ratio, *MMP-9* matrix metalloproteinase 9

Based on the ER status–related differences we have observed at the association of these macrophage markers with survival, we have tested whether breast cancer subtypes are associated with different TAM subpopulations. In cohort A, expression of all three biomarkers (CD68, CD163, and MMP-9) was higher in ER^−^ tumors (Additional file 3: Tables S1, S2, and Additional file 2: Figure S5 E–H). In ER^+^ tumors (cohort A), MMP-9 was expressed mainly in CD68^+^/CD163^+^ TAMs (*P* = 0.007) compared with CD68-only TAMs; in TNBC cohort B, it was less expressed in CD163^+^/CD68^+^ TAMs and more widely distributed in all CD68^+^ macrophages (*P* <0.0001, Additional file 2: Figure S5). MMP-9 expression was significantly higher in ER^−^ tumors than in ER^+^ tumors of cohort A (CD68^+^*P* = 0.0001) (CD68^+^/CD163^+^*P* = 0.001) (Additional file 2: Figure S5, G, H). MMP-9 was also inversely correlated with progesterone receptor status in CD68^+^/CD163^+^ TAMs (chi-squared, *P* = 0.03). CD163 expression was also associated with lymph node status (*P* = 0.001). In TNBC cohort B, high MMP-9 expression was associated with higher grade in all CD68^+^ (*P* = 0.009) and CD163^+^/CD68^+^macrophages (*P* = 0.01). This association was not evidenced when CD68 or CD163 was compared with these clinicopathological parameters (Additional file 3: Tables S3, S4). No significant correlation was evidenced with HER2 status (not shown) for any of the TAM markers.

### Comparison of protein *in situ* detection with METABRIC data

**Fig. 4:**
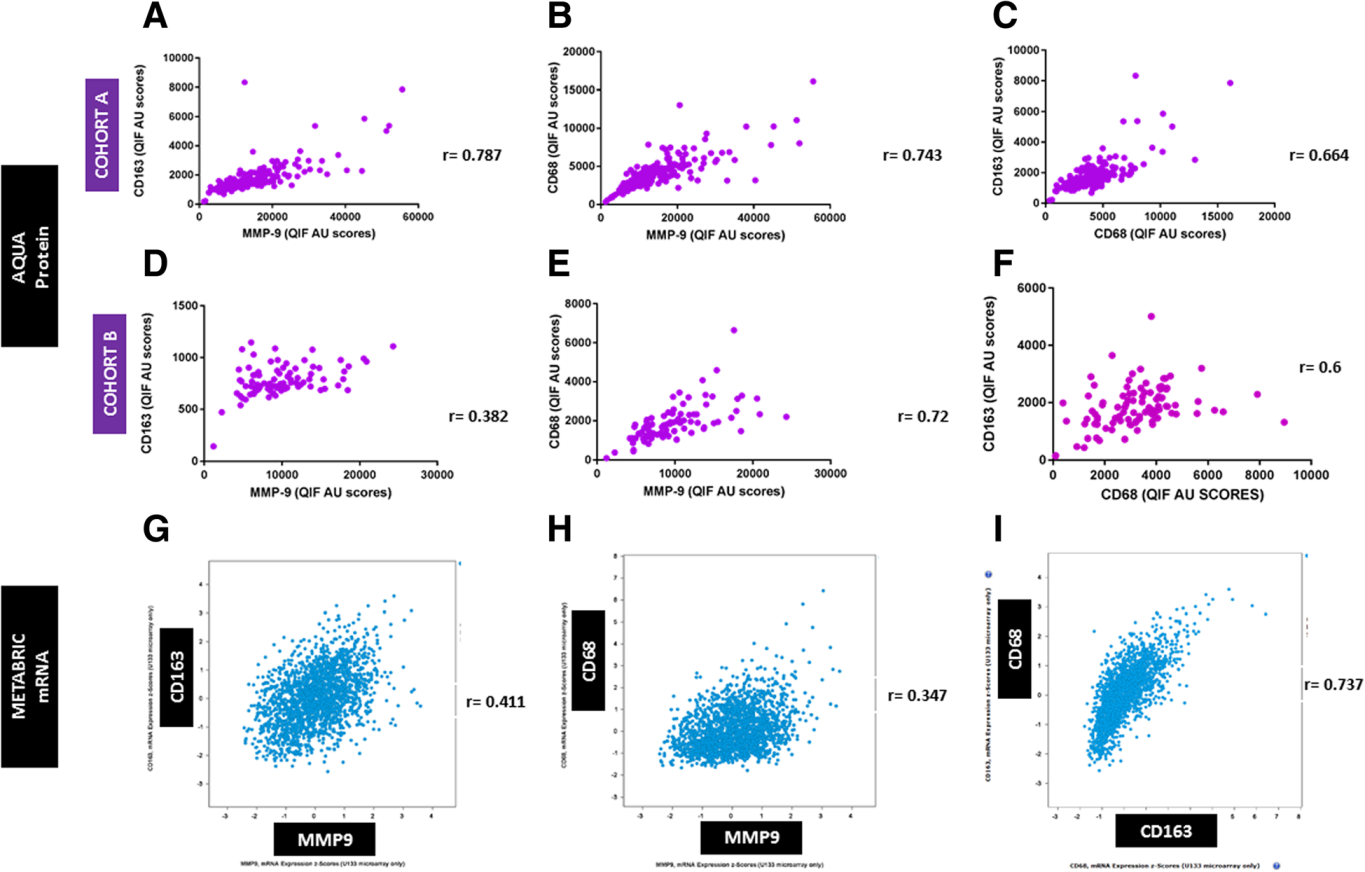
Comparison of AQUA protein with METABRIC mRNA expression data. AQUA protein detection and Spearman correlation of tumor-associated macrophage (TAM) biomarker quantitative immunofluorescence (QIF) scores in cohort a (upper panel, a–c) and triple-negative breast cancer (TNBC) cohort B (middle panel, d–f). **a** Correlation of matrix metalloproteinase 9 (MMP-9) and CD163. **b** Correlation of MMP-9 and CD68. **c** Correlation of CD163 and CD68. **d** Correlation of MMP-9 and CD163. **e** Correlation of MMP-9 and CD68. **f** Correlation of CD163 and CD68. At the lower panel correlations of TAM biomarkers, mRNA z-scores from the METABRIC study are shown. **g** Correlation of MMP-9 and CD163 mRNA. **h** Correlation of MMP-9 and CD68 mRNA. **i** Correlation of CD163 and CD68 mRNA. Abbreviation: *AU* arbitrary units of fluorescence

CD68, CD163, and MMP-9 mRNA expression levels in the METABRIC study were correlating, but reached significance for only the MMP-9–CD163 pair (*P* <0.001, Spearman *r* = 0.41) and CD68-CD163 pair (*P* <0.001, Spearman *r* = 0.737) (Fig. 4). In our breast cancer cohorts, protein measurement of these biomarkers by QIF displayed similar patterns (Fig. 4, protein expression of each marker in all fields of view, without co-localization to assimilate information retrieved from mRNA data) but with significant correlations for all three pairs (MMP-9–CD163 *P* <0.001, Spearman *r* = 0.78; MMP-9–CD68 *P* <0.001, Spearman *r* = 0.8; and CD68-CD163 *P* <0.001, Spearman *r* = 0.664). mRNA levels of all three markers were higher in ER^−^ tumors; MMP-9 mRNA, in particular, was higher in ER^−^ tumors (ER status by immunohistochemistry and ER transcript) and non-luminal tumors, grade 3, and premenopausal patients. Finally, OS analysis of the macrophage markers’ mRNA individually, stratified by ER status, showed similar trends as the ones we observed by QIF for MMP-9 and CD68 but not for CD163. More precisely, high MMP-9 mRNA levels (optimal cut point defined by X-tile software after multiple comparison correction) were associated with shorter survival only in ER^+^ patients (*P* = 0.006), confirming our QIF data. CD68 mRNA had no significant association with OS in ER^+^ (*P* = 0.11) but exhibited a non-significant trend for impaired survival in ER^−^ cases (*P* = 0.053), approaching the significant association with worse outcome we observed in ER^+^ patients by QIF. However, high CD163 mRNA levels were associated with shorter OS in ER^+^ patients (*P* = 0.03) but the opposite association was observed by QIF (longer survival with higher CD163 levels).

## Discussion

Breast cancer intervention strategies have been traditionally tumor cell–centered. Recent approaches endorse a paradigm shift encompassing interactions between tumor cells and microenvironment aiming to overcome resistance to treatment but also improve efficiency and long-term effects of therapeutic approaches. Introduction of immune-related markers to breast cancer management, such as TILs, has been proven to be a useful predictive tool, especially for achievement of pathologic complete response following treatment [3–5, 9, 29–31]. Immunotherapy (especially PD-axis targeting) has revolutionized the management of many solid tumors, and recent data from early and advanced stage breast cancer trials are encouraging [1, 2]. Hence, in breast cancer, compared with other neoplasms, PD-L1 expression levels are relatively low (about 15–30% of cases) [7], and lymphocyte infiltration in most breast tumors is modest [6, 9]. Therefore, in addition to manipulation of the adaptive immune system, inclusion of the innate arm of the immune system, where TAMs play an important role, might result in better tumor management.

Conventionally, macrophage subpopulations have been described as either classically activated (M1, pro-inflammatory, or tumoricidal) or alternatively activated (M2 specialized to suppress inflammation) [15]. This M1/M2 subgrouping underrepresents the diverse functional spectrum acquired in response to changing environmental stimuli and is not strictly indicative of their anti-tumor or immune-suppressive role. Although both MMP-9 and CD163 have been traditionally related with M2-phenotype, here we show that they do not always correlate with worse prognosis, as previously reported in breast cancer [20–22, 32–34]. So far, non-small cell lung, prostate, and colorectal cancer are the notable exemptions where intense TAM infiltration is associated with better outcome [23]. We also show that ER status is an important determinant of the association of these TAM markers’ expression with outcome. Interestingly, although all three—CD68, CD163, and MMP-9—are preferentially expressed in ER^−^ and non-luminal tumors, both at protein and mRNA level, MMP-9 is associated with worse outcome only in ER^+^ tumors. Although this initially appears to be a paradox, it could be indicative of bypass mechanisms that activate the expression of the protein in TAMs of some ER^+^ tumors or induce the recruitment of certain subclasses of TAMs. Indeed, we show that, in ER^+^ tumors, MMP-9 is found mostly in CD163^+^ TAMs but that, in ER^−^ tumors, it was higher in all CD68^+^ macrophages. This pattern could be indicative of recruitment of specific TAM subtypes or induction of TAM reprogramming (phenotypical and functional polarization) by different tumor cell subtypes, as previously shown in *in vitro* [35–38] and breast cancer tissue [39] studies. It could also underline the importance of further exploration of how this TAM pattern could be affected by established treatment modalities (especially endocrine therapy in ER^+^ tumors, chemotherapy/radiotherapy, or immune therapies) or could modulate response to them and how this could be exploited to optimize responses or manipulate resistance [14–16]. We could not establish an association of CD68, CD163, or MMP-9 with HER2 status, which could be partially attributed to the low number of positive 3+ cases (*n* = 20, 5%) and high number of unknown/equivocal cases (*n* = 106, 26.6%).

In previous studies, TAMs have been evaluated subjectively by semi-quantitative chromogenic methods and several antibodies with different antigen retrieval, titer, and detection systems [19–23]. Consequently, the quantitative approach we use in this work is not directly comparable to that of previous reports. Our discrepancy with previous reports could be attributed to the single biomarker methodology used by other groups and their limitations to detect the M1/M2 dichotomy to capture the net effect of TAM biomarkers on patient survival. Variability in definitions, outcomes, measurements, experimental procedure, antibody titration, validation, and concentration may contribute to heterogeneity between studies. Our data suggest that determination of expression levels of more than one TAM biomarker, identification of co-expression or mutual exclusivity, spatial context (co-localization), and hormone receptor status are important for investigation of their impact on patient prognosis. This could also partially explain the discrepancy we observed in survival evaluation of TAM marker expression between mRNA and QIF. The most representative example is the one of CD163, the hallmark of M2-like phenotype, which would conventionally be expected to represent a worse outcome prognosticator. However, this was not the case when assessed by QIF and we mostly attribute this to the fact that the levels of other proteins, such as MMP-9, should be co-assessed to better reflect the function of TAMs in certain tumors.

There are a number of limitations to this work. Perhaps most significantly, it is based on a retrospective assessment of two, small, single-institution, breast cancer cohorts, both of which are heterogeneously treated. We show only OS data since do not have adequate recurrence data to assess the predictive profile of these biomarkers. Another limitation is that we examined only two M2 markers (MMP-9 and CD163) of the many described that could be co-expressed in these specimens and affect outcome or subclassification. Finally, our cases were represented in TMA format, which may induce under- or over-representation of the marker levels because of tumor heterogeneity. However, the comparable results in most of the co-expression seen in the METABRIC dataset using mRNA measurements in whole-tissue section tumor samples support the validity of our findings.

## Conclusions

TAM measurement and related evaluation criteria for companion diagnostics are yet to be established. Objective *in situ* TAM subclassification, using multiplexed assays based on validated antibody panels, reveals TAM diversity that is expected but not previously shown using *in situ* methods. These methods appear to be useful to understand the functional status of macrophages and may be useful in the future for companion diagnostic testing as drugs are developed that target this cell type, such as MMP-9 targeting compounds. Our findings, identified ER^+^ tumors with high levels of MMP-9/CD163 co-expression as the potential target breast cancer group that could benefit from an MMP-9 targeting modality.

## Additional files

Additional file 1: Antibody validation. (DOCX 23 kb)

Additional file 2: **Figure S1.** CD68 antibody validation. (A) Comparison of two CD68 monoclonal antibodies (PG-M1, SP251). Regression (R^2^) of QIF scores in breast TMA. (B) Overlayed images (PG-M1/left, SP251/right (CD68/red/Cy5), (CK/green/Cy3), (DAPI/blue). Bar = 100 μm. (C) Stained myeloid cells FFPE-pellets (PG-M1/CD68/red/Cy5, DAPI/blue). (D) PG-M1 QIF scores of transfected U937 cells (MMP-9 scramble/siRNA). **Figure S2.** CD163 antibody validation. (A) IL-10-induced CD163 expression in U937 cells. (Mann–Whitney, mean ± SEM). (B) QIF overlayed images of U937 cells FFPE-pellets (CD163/red/Cy5, DAPI/Blue). (C) M-CSF-induced CD163 expression in U937 cells (Mann–Whitney, mean ± SEM). (D) QIF overlayed images of U937 pellets (CD163/red/Cy5, DAPI/blue). (E) Regression of two CD163 antibodies (CD163-L-U, D6U1J) QIF scores in breast cancer TMA. **Figure S3.** MMP-9 antibody validation-Comparison of (DX6O3H-XP, G657) antibodies. (A) Regression of MMP-9+/CD68+ QIF scores in breast cancer TMA. (B) Overlayed images (CD68/green, MMP-9/red, DAPI/blue) (G657/left, DX6O3H-XP/right). (C) QIF images of cell line FFPE-pellets (MMP-9/red/Cy5, DAPI/blue) (DX6O3H-XP/upper, G657/lower. Bar = 200 μm) (lower). **Figure S4.**, left), middle right MMP-9 silencing (U937). (A) Representative monochrome (MMP-9/Cy5/left, nuclei/DAPI/middle, merged/right) images of U937 cells transfected with scramble/upper, MMP-9 siRNA A/middle, MMP-9 siRNA B/lower. Bar = 200 μm. (Β) ΜΜP-9 QIF scores of U937 cells transfected with scramble/siRNA. Mean ± SEM. **Figure S5.** quantitative immunofluorescence (QIF) scores. Distribution of CD68, CD163, and MMP-9 QIF scores. CD68/Red and CD163/Yellow in cohort A (A) and TNBC cohort B (B). MMP-9 QIF scores in CD68+/blue and CD163+/red, cells (C, cohort A, and D, Cohort B). (E) MMP-9 QIF scores among all CD68+ and CD68+/CD163+ TAMs in cohort A. (F) Comparison of MMP-9 QIF scores among all CD68+ and CD68+/CD163+ TAMs in TNBC cohort B. (G) Comparison of MMP-9 QIF scores among all CD68+ macrophages per ER status (cohort A). (H) Comparison of MMP-9 QIF scores among CD163+/CD68+TAMs per ER status (cohort A). Mann–Whitney test, mean ± SEM. Abbreviation: AU arbitrary units of fluorescence. (PDF 1210 kb)

Additional file 3: **Table S1.** Clinicopathological characteristics according to CD68 and CD163 quantitative immunofluorescence (QIF) scores in cohort A. **Table S2.** Clinicopathological characteristics according to matrix metalloproteinase 9 (MMP-9) quantitative immunofluorescence (QIF) scores in CD68 and CD163 compartments in cohort A. **Table S3.** Clinicopathological characteristics according to CD68 and CD163 quantitative immunofluorescence (QIF) scores in cohort B. **Table S4.** Clinicopathological characteristics according to matrix metalloproteinase 9 (MMP-9) quantitative immunofluorescence (QIF) scores in CD68 and CD163 compartments in cohort B. (PDF 205 kb)

## Abbreviations

CD: Cluster of differentiation
Cy: Cyanine
DAPI: 4,6-Diamidino-2-phenylindole
ER: Estrogen receptor
H&E: Hematoxylin-eosin
HRP: Horseradish peroxydase
MMP-9: Matrix metalloproteinase 9
OS: Overall survival
QIF: Quantitative immunofluorescence
TAM: Tumor-associated macrophage
TIL: Tumor-infiltrating lymphocyte
TMA: Tissue microarray
TNBC: Triple-negative breast cancer
