# Supplementary material for: CD68, CD163, and matrix metalloproteinase 9 (MMP-9) co-localization in breast tumor microenvironment predicts survival differently in ER-positive and -negative cancers
Source: Breast Cancer Res. 2018 Dec 17;20:154. doi: 10.1186/s13058-018-1076-x (PMC6298021; doi:10.1186/s13058-018-1076-x)
Supplement: Supplementary file 1 — Antibody validation. (DOCX 23 kb) [file 13058_2018_1076_MOESM1_ESM.docx]

**Additional file 1**

**Antibody validation**

To determine specificity of our antibodies, we have performed a series of genetic and antibody-independent assays, following established antibody validation criteria (1, 2). Briefly, we have tested constitutive expression in cell lines of myeloid and epithelial origin, as well as index TMAs (tonsil/lymph nodes and breast) and then compared with genetically modified cell lines, by silencing mRNA of the target proteins (MMP-9, CD68) or induction of expression by means of cytokines and growth factors (IL-10, CSF-1R) for CD163. Two different antibodies for each TAM biomarker were compared.

Open-access databases (Expression Atlas European bioinformatics institute EMBL-EBI, https://www.ebi.ac.uk/gxa/home and Cell Line Atlas cell line atlas - The Human Protein Atlas, www.proteinatlas.org/cell) were reviewed for cell line mRNA and protein expression of MMP-9, CD68, CD163). SW480 colon cell line was selected for MMP-9 negative control due to low mRNA expression. Myeloid cell lines KG-1, KG-1A and U-937 were selected as CD68, CD163 and MMP-9 expressers. All cell lines were obtained from the American Type Culture Collection (ATCC) and maintained as recommended.

1. Cell FFPE pellets were also prepared in order to follow the same antigen retrieval as in our TMAs. Briefly, cells were washed twice in PBS, collected and kept in formalin overnight at 4^o^C. Cells were then resuspended and rinsed three times in PBS before being washed twice in 80% EtOH. Cell pellets were then spun at 12,000 RPM and embedded in 2.2% melted agarose in PBS. Agarose-embedded pellets were incubated in 70% EtOH overnight and then sequentially dehydrated with one-hour incubations of 90% EtOH and 100% EtOH, two one-hour xylene washes, and submerged in molten paraffin for two hours before embedding.
2. **U937 Cell Line Silencing**

Cells were kept in Gibco™ RPMI Medium (ThermoFisher Scientific, USA), supplemented with 20% FBS, Penicillin-Streptomycin (10,000 U/mL) at 37^o^C, 5% CO2. When they reached 70% confluency, they were transferred to Optimem-Low Serum Medium (ThermoFisher Scientific, USA), transfected with 1 nM and 10nM of MMP-9 and scrambled siRNA Trilencer-27 Human siRNA (OriGene Technologies, Inc, USA) with RNAiMax (ThermoFisher Scientific, USA), following manufacturer’s instructions. After 48h incubation, cells were collected and replated on Poly-L-Lysine (Sigma) coated coverslips in duplicates. They were washed twice in PBS (Life Technologies) and fixed in 4% Paraformaldehyde (ThermoFisher Scientific, USA) supplemented with sucrose for 10 minutes. Then, following double wash in PBS they were permeabilized in 0.25% Triton x100-PBS for 10 minutes. After a double wash in PBS, a blocking in 2% BSA-PBS for 1 hour at room temperature. Primary antibody against MMP-9 (rabbit monoclonal, clone DX6O3H-XP, Cell Signaling Technologies, 0.58 μg/ml) was added overnight at 4^o^C, in light protected chamber. Then, after 2 washes of 1% tween-PBS and one in PBS 5 minutes each, secondary antibodies were added using Rabbit Envision Neat for 1hr at room temperature. After PBS-tween /PBS wash, Cy5-tyramide (10μL Cy5 tyramide in 490 Amplification buffer) was added for 10 minutes. Finally, after a last PBS-tween /PBS wash mounting was performed with Prolong Gold – DAPI (Life Technologies). Quantitative immunofluorescence microscopy and EVOS-FL (LIFE Technologies) inverted microscope were used for assessment of protein expression for MMP-9 silencing.

For CD68 silencing 1nmol and 10nmol of CD68-siRNA and scrambled siRNA Trilencer-27 Human siRNA (OriGene Technologies, Inc, USA) with Lipofectamine 2000 (ThermoFisher Scientific, USA), following manufacturer’s instructions. Cells were transfected using Lipofectamin® 2000, 1 and 10 nmol of each siRNA for 24 hours, and then cultured with low FBS OPTI-MEM medium for additional 24 hours. Control and knocked-down cells were used fresh for protein extraction and immunoblotting; or fixed in 10% neutral buffered formalin for 8-12 h and embedded in paraffin for CD68 quantification by QIF.

**Induction of CD163**

U937 cells at a density of 10^5^/mL were treated with IL-10 (R&D, Biotechne, MN, USA) at 10 and 25ng/mL for 24h to polarize them towards M2-like CD163+ in flasks and poly-lysine L coated coverslips. They were then collected and followed staining protocol with CD163 as mentioned above for MMP-9 and CD68.

Cells were also treated with M-CSF (R&D, Biotechne, MN, USA) at 50ng/mL and collected after 5 days. CD163 staining was performed as mentioned above.

All conditions were tested in triplicates.

**Comparison of MMP-9 antibodies**

Two rabbit MMP-9 antibodies, the monoclonal DX6O3H-XP (0.58 μg/ml, Cell Signaling Technologies) and the polyclonal G657 (Cell Signaling Technologies, 1/1000) were compared in a breast cancer index TMA and sections from FFPE pellets of three myeloid (U937, KG-1A, HL-60) and the colon SW480 adenocarcinoma cell line.

Serial section slides were used for analyzing the staining and protein expression patterns for the two markers. Briefly, TMA and cell line slides were baked overnight at 60°C and then soaked in xylene twice for 20 minutes each. Slides were rehydrated in two 1-minute washes in 100% ethanol followed by one wash in 70% ethanol and finally rinsed in streaming tap water for 5 minutes. Antigen retrieval was performed in EDTA buffer, pH 8 for PH8 in the PT-Link module from LabVision for 20 min at 97°C in a pressure-boiling container. Blocking was performed with 0.3% bovine serum albumin in 0.05% tween solution for 30 minutes after antigen retrieval. Each of the MMP-9 clones was used for target detection and was combined with either 1:100 pan-cytokeratin antibody (Dako) or CD68 (clone PG-M1, Dako, 0.3 μg/ml) in 0.3% BSA in TBST and incubated overnight at 4°C. Primary antibodies were followed by incubation with Alexa 546–conjugated goat anti-mouse (Molecular Probes, Eugene, OR, USA) diluted 1:100 in rabbit EnVision reagent (Dako) for 1 hour. Signal was amplified with Cyanine 5 (Cy5) directly conjugated to tyramide (Perkin-Elmer, Waltham, MA, USA) at 1:50 dilution. ProLong mounting medium (ProLong Gold; Molecular Probes) with 4,6-diamidino-2-phenylindole (DAPI) was used to stain nuclei.

**Comparison of CD68 Antibodies.**

Two monoclonal CD68 antibodies, the mouse PG-M1 (0.15 μg/ml, DAKO) and the rabbit SP251 (0.32 μg/ml Spring) were compared in a breast cancer index TMA and sections from FFPE pellets of myeloid (U937, KG-1A, KG-1 and HL-60) cell lines.

Serial section slides were used for analyzing the staining and protein expression patterns for the two markers, as described above.

**Comparison of CD163 Antibodies.**

Two monoclonal CD163 antibodies, the mouse CD163-L-U (0.006 μg/ml, Leica, Novocastra) and the rabbit D6UIJ (0.088 μg/ml Cell Signaling Technology) were compared in a breast cancer index TMA.

Serial section slides were used for analyzing the staining and protein expression patterns for the two markers, as described above.

1. Bordeaux J, Welsh A, Agarwal S, Killiam E, Baquero M, Hanna J, et al. Antibody validation. Biotechniques. 2010;48:197-209.

2. Uhlen M, Bandrowski A, Carr S, Edwards A, Ellenberg J, Lundberg E, et al. A proposal for validation of antibodies. Nat Methods. 2016;13:823-7.
