# Supplementary material for: CD68, CD163, and matrix metalloproteinase 9 (MMP-9) co-localization in breast tumor microenvironment predicts survival differently in ER-positive and -negative cancers
Source: Breast Cancer Res. 2018 Dec 17;20:154. doi: 10.1186/s13058-018-1076-x (PMC6298021; doi:10.1186/s13058-018-1076-x)

Figure S1. CD68 antibody validation (PG-M1, SP251). **(A)** Regression (R2) of QIF scores in breast TMA. **(B)** Overlayed images (PG-M1/left, SP251/right), (CD68/red/Cy5), (CK/green/Cy3), (DAPI/blue). Bar = 100 µm. **(C)** Stained myeloid cells FFPE-pellets (PG-M1/CD68/red/Cy5, DAPI/blue). **(D)** PG-M1 QIF scores of transfected U937 cells (MMP-9 scramble/siRNA). Mean ± SEM.

Supplementary Figure S1

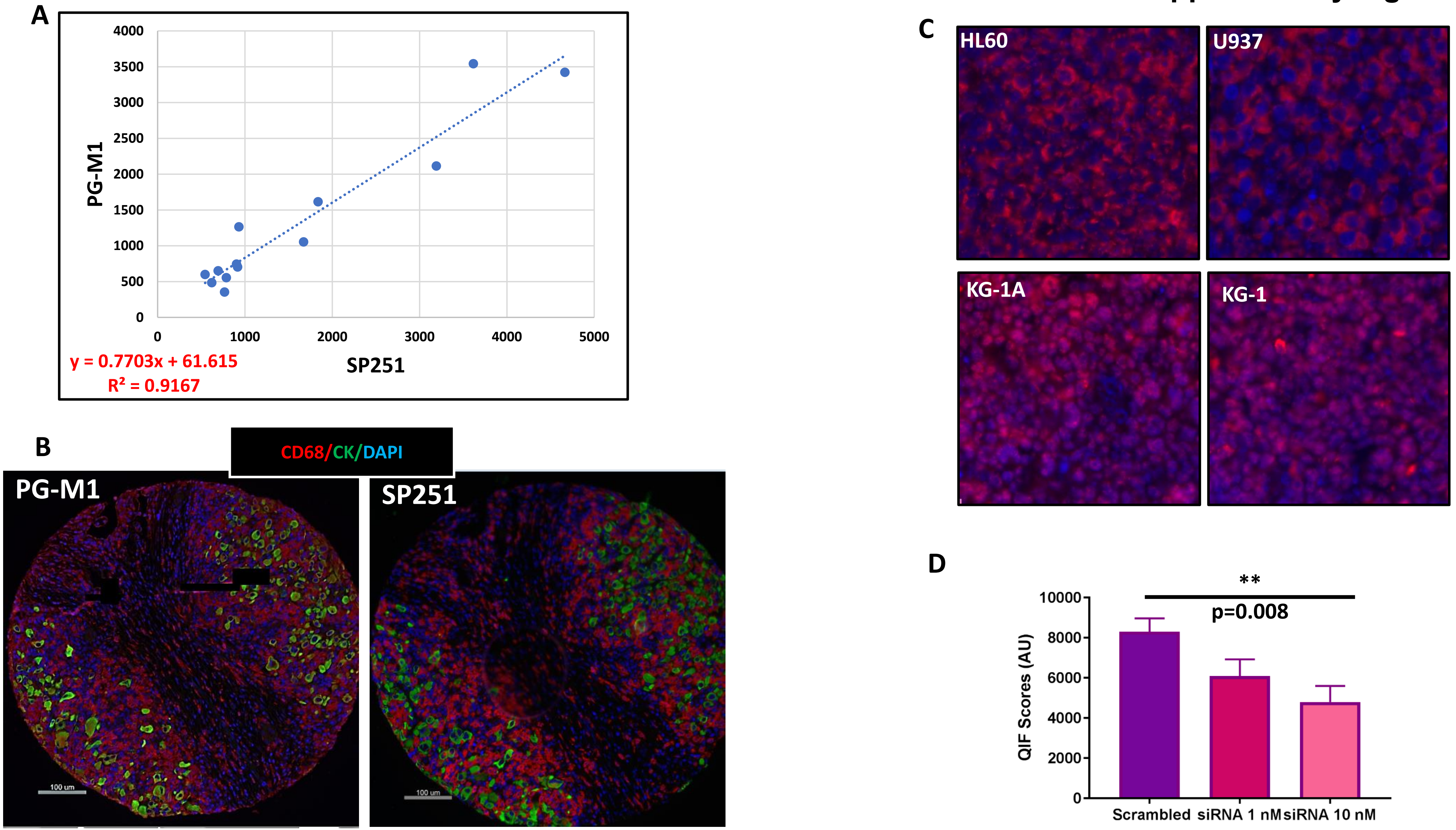

Figure S2. CD163 antibody validation. **(**A**)** IL-10-induced CD163 expression in U937 cells. (Mann–Whitney, mean ± SEM). **(**B**)** QIFoverlayed images of U937 cells FFPE-pellets (CD163/red/Cy5, DAPI/Blue). **(**C**)** M-CSF-induced CD163 expression in U937 cells (Mann–Whitney, mean ± SEM). **(**D**)** QIF overlayed images of U937 pellets (CD163/red/Cy5, DAPI/blue). **(**E**)** Regression of two CD163 antibodies (CD163-L-U, D6U1J) QIF scores in breast cancer TMA.

Supplementary Figure S2

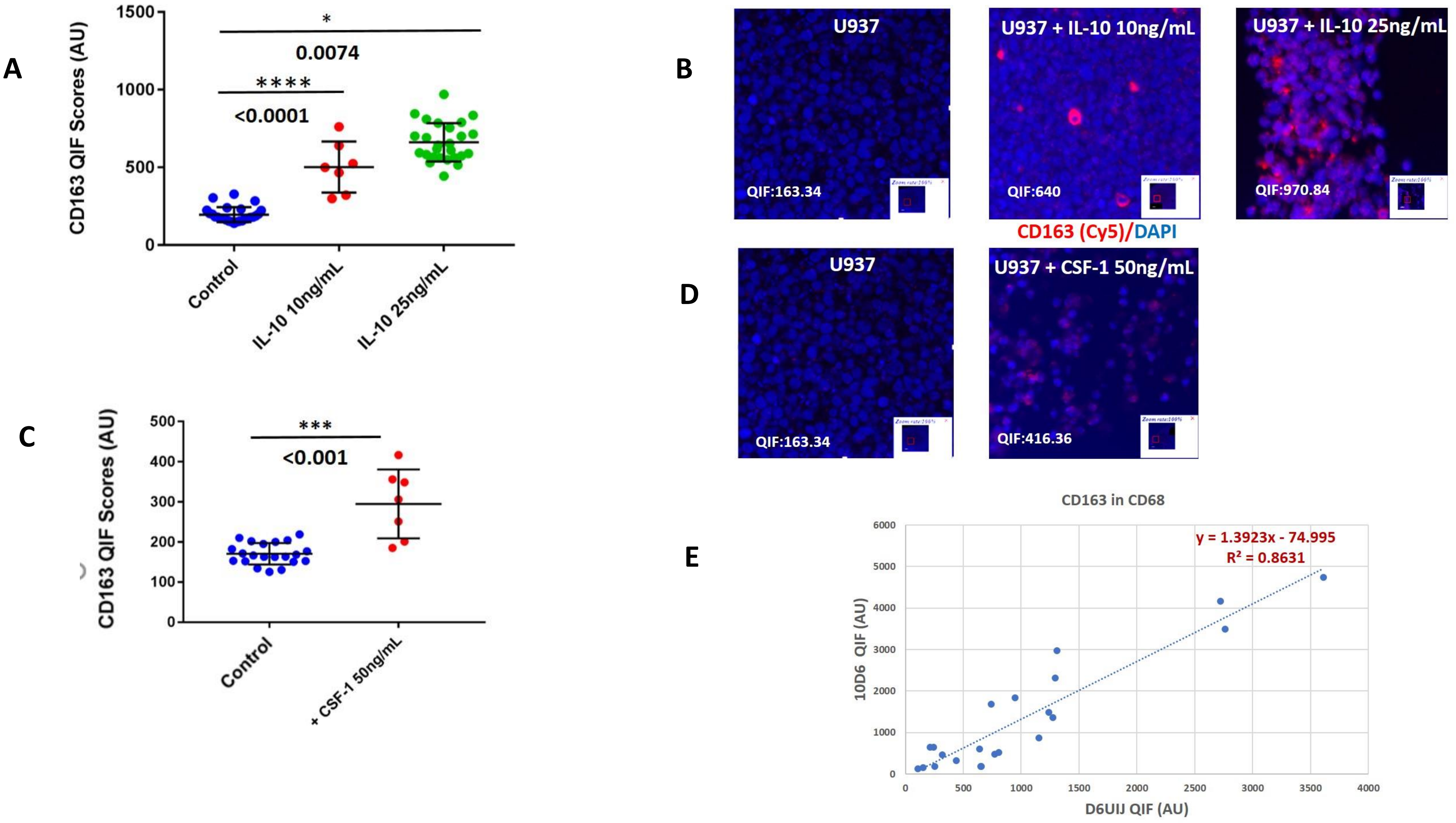

Figure S3. MMP-9 antibody validation-Comparison of (DX6O3H-XP, G657) antibodies. **(**A**)** Regression of MMP-9+/CD68+ QIF scores in breast cancer TMA. **(**B**)** Overlayed images (CD68/green, MMP-9/red, DAPI/blue) (G657/left, DX6O3H-XP/right). **(**C**)** QIF images of cell line FFPE-pellets (MMP-9/red/Cy5, DAPI/blue) (DX6O3H-XP/upper, G657/lower. Bar = 200 μm.

Supplementary Figure S3

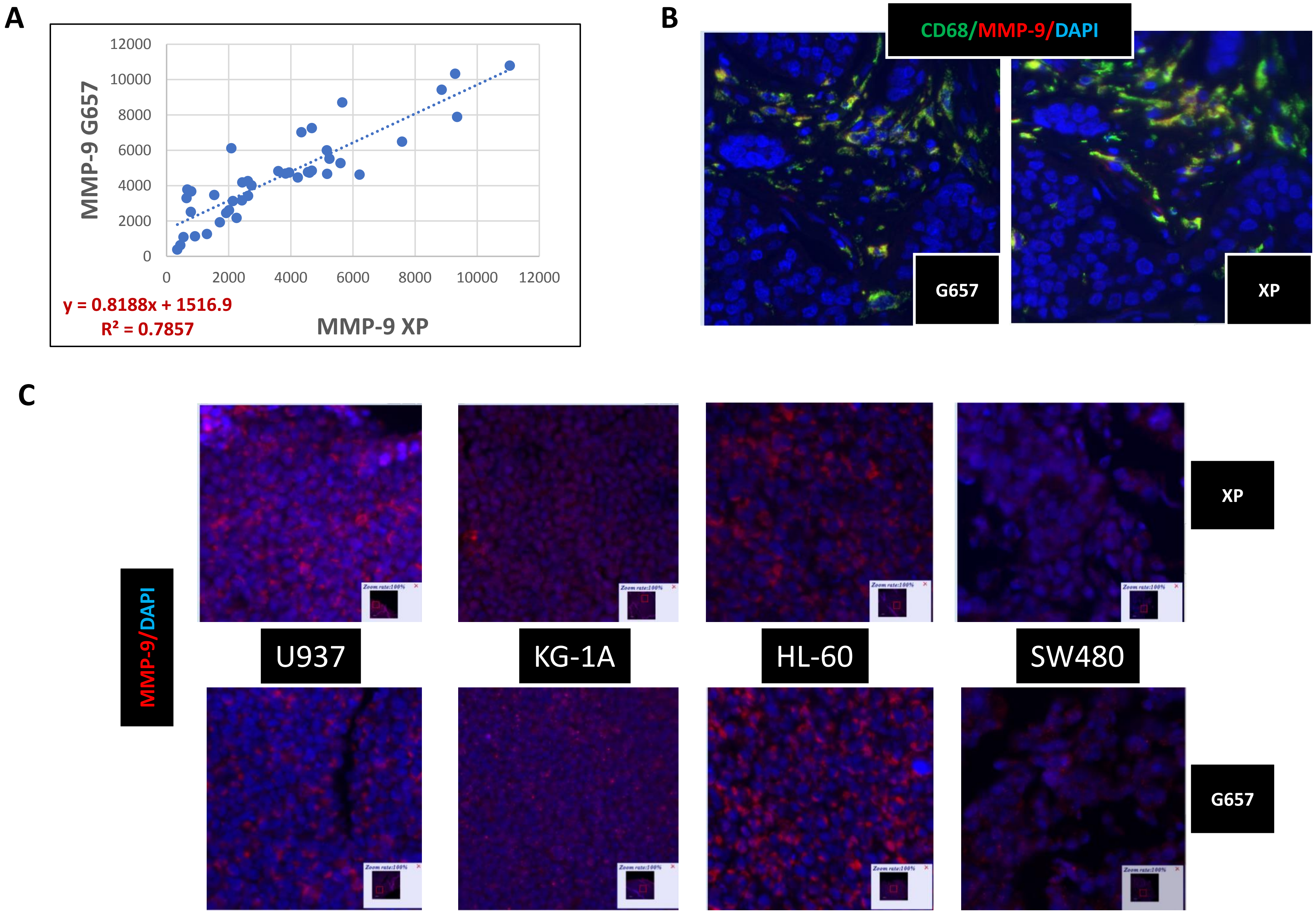

Figure S4. MMP-9 silencing (U937). **(**A**)** Representative monochrome (MMP-9/Cy5/left, nuclei/DAPI/middle, merged/right) images of U937 cells transfected with scramble/upper, MMP-9 siRNA A/middle, MMP-9 siRNA B/lower. Bar = 200 µm. **(**B**)** MMP-9 QIF scores of U937 cells transfected with scramble/siRNA. Mean ± SEM.

Supplementary Figure S4

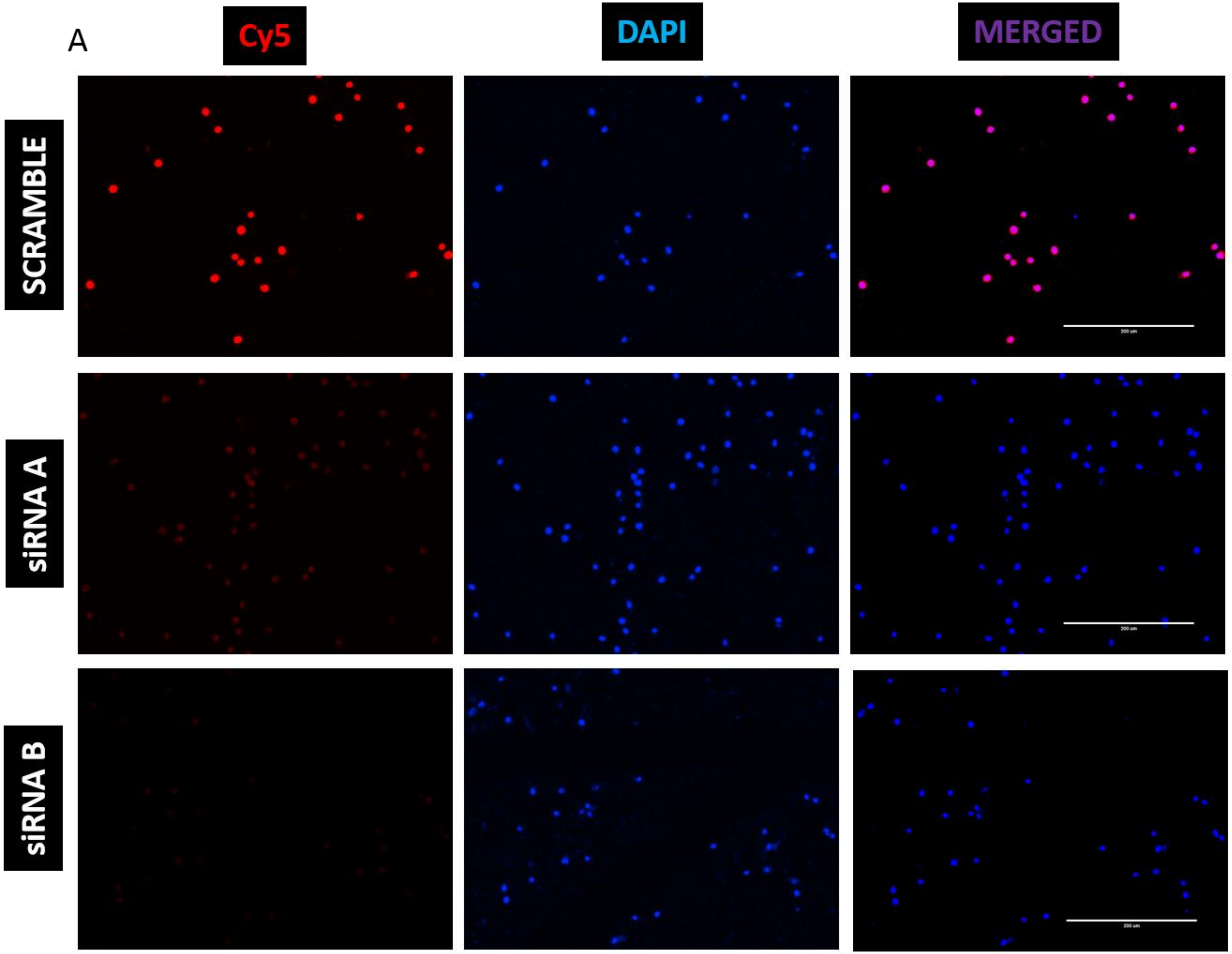

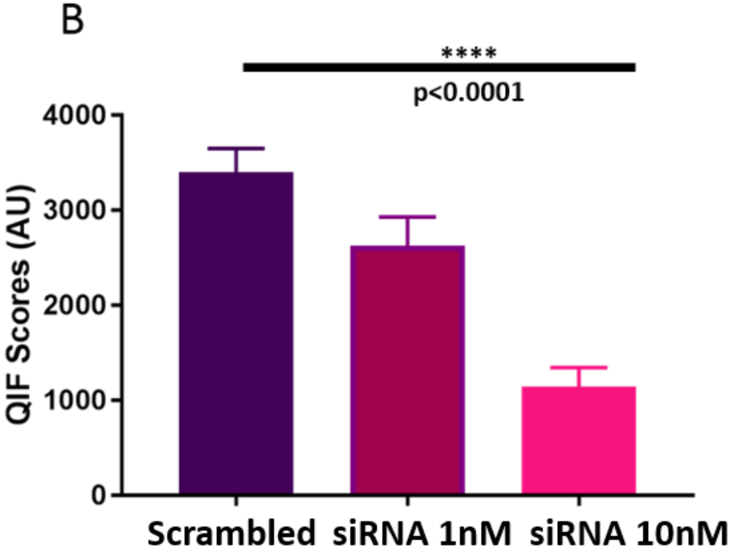

Figure S5. Distribution of CD68, CD163, and MMP-9 QIF scores. CD68/Red and CD163/Yellow in cohort A **(**A**)** and TNBC cohort B **(**B**)**. MMP-9 QIF scores in CD68+/blue and CD163+/red, cells (C, cohort A, and D, Cohort B). **(**E**)** MMP-9 QIF scores among all CD68+ and CD68+/CD163+ TAMs in cohort A. **(**F**)** Comparison of MMP-9 QIF scores among all CD68+ and CD68+/CD163+ TAMs in TNBC cohort B. **(**G**)** Comparison of MMP-9 QIF scores among all CD68+ macrophages per ER status (cohort A). **(**H**)** Comparison of MMP-9 QIF scores among CD163+/CD68+TAMs per ER status (cohort A). Mann–Whitney test, mean ± SEM. Abbreviation: AU arbitrary units of fluorescence.

Supplementary Figure S5

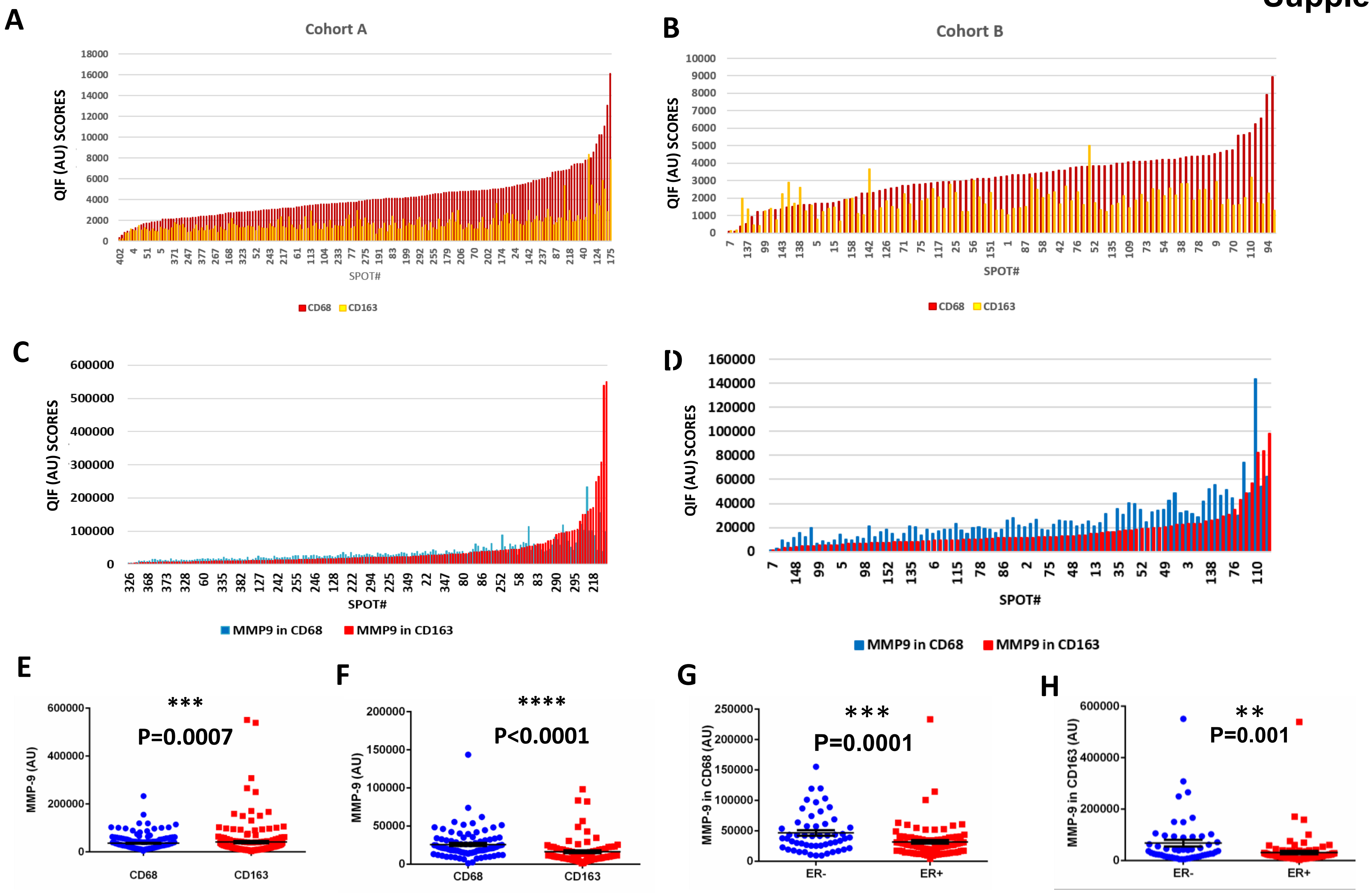

Supplement: Supplementary file 2 — Figure S1. CD68 antibody validation. (A) Comparison of two CD68 monoclonal antibodies (PG-M1, SP251). Regression (R2) of QIF scores in breast TMA. (B) Overlayed images (PG-M1/left, SP251/right (CD68/red/Cy5), (CK/green/Cy3), (DAPI/blue). Bar = 100 μm. (C) Stained myeloid cells FFPE-pellets (PG-M1/CD68/red/Cy5, DAPI/blue). (D) PG-M1 QIF scores of transfected U937 cells (MMP-9 scramble/siRNA). Figure S2. CD163 antibody validation. (A) IL-10-induced CD163 expression in U937 cells. (Mann–Whitney, mean ± SEM). (B) QIF overlayed images of U937 cells FFPE-pellets (CD163/red/Cy5, DAPI/Blue). (C) M-CSF-induced CD163 expression in U937 cells (Mann–Whitney, mean ± SEM). (D) QIF overlayed images of U937 pellets (CD163/red/Cy5, DAPI/blue). (E) Regression of two CD163 antibodies (CD163-L-U, D6U1J) QIF scores in breast cancer TMA. Figure S3. MMP-9 antibody validation-Comparison of (DX6O3H-XP, G657) antibodies. (A) Regression of MMP-9+/CD68+ QIF scores in breast cancer TMA. (B) Overlayed images (CD68/green, MMP-9/red, DAPI/blue) (G657/left, DX6O3H-XP/right). (C) QIF images of cell line FFPE-pellets (MMP-9/red/Cy5, DAPI/blue) (DX6O3H-XP/upper, G657/lower. Bar = 200 μm) (lower). Figure S4., left), middle right MMP-9 silencing (U937). (A) Representative monochrome (MMP-9/Cy5/left, nuclei/DAPI/middle, merged/right) images of U937 cells transfected with scramble/upper, MMP-9 siRNA A/middle, MMP-9 siRNA B/lower. Bar = 200 μm. (Β) ΜΜP-9 QIF scores of U937 cells transfected with scramble/siRNA. Mean ± SEM. Figure S5. quantitative immunofluorescence (QIF) scores. Distribution of CD68, CD163, and MMP-9 QIF scores. CD68/Red and CD163/Yellow in cohort A (A) and TNBC cohort B (B). MMP-9 QIF scores in CD68+/blue and CD163+/red, cells (C, cohort A, and D, Cohort B). (E) MMP-9 QIF scores among all CD68+ and CD68+/CD163+ TAMs in cohort A. (F) Comparison of MMP-9 QIF scores among all CD68+ and CD68+/CD163+ TAMs in TNBC cohort B. (G) Comparison of MMP-9 QIF scores among all CD68+ macrophages per ER s [file 13058_2018_1076_MOESM2_ESM.pdf]
