# Supplementary material for: CD68, CD163, and matrix metalloproteinase 9 (MMP-9) co-localization in breast tumor microenvironment predicts survival differently in ER-positive and -negative cancers
Source: Breast Cancer Res. 2018 Dec 17;20:154. doi: 10.1186/s13058-018-1076-x (PMC6298021; doi:10.1186/s13058-018-1076-x)
Supplement: Supplementary file 3 — Table S1. Clinicopathological characteristics according to CD68 and CD163 quantitative immunofluorescence (QIF) scores in cohort A. Table S2. Clinicopathological characteristics according to matrix metalloproteinase 9 (MMP-9) quantitative immunofluorescence (QIF) scores in CD68 and CD163 compartments in cohort A. Table S3. Clinicopathological characteristics according to CD68 and CD163 quantitative immunofluorescence (QIF) scores in cohort B. Table S4. Clinicopathological characteristics according to matrix metalloproteinase 9 (MMP-9) quantitative immunofluorescence (QIF) scores in CD68 and CD163 compartments in cohort B. (PDF 205 kb) [file 13058_2018_1076_MOESM3_ESM.pdf]

Table S1
Clinicopathological characteristics according to CD68 and CD163 QIF scores in cohort A

COHORT A	CD68 Low	CD68 High	p-value	CD163 Low	CD163 High	p-value
ER			0.95			**0.002**
ER Negative	34	38		16	37	
ER Positive	60	66		51	40	
PR			0.26			0.05
PR Negative	34	48		20	35	
PR Positive	55	56		45	40	
HER2			0.77			0.09
HER2 Negative	24	38		19	27	
HER2 Positive	6	8		1	7	
Size			**0.08**			0.09
<2cm	73	62		48	43	
>2 cm	34	47		26	37	
Age			0.5			0.09
<50 Years	37	44		24	34	
50 or >50 years	80	69		61	48	
LN Status			0.16			
LN neg	56	65		68	64	**0.001**
LN pos	9	23		1	11	
Grade			0.33			0.53
1or 2	26	40		19	30	
3	21	46		15	31	
Recurrence			0.11			0.11
Yes	86	93		86	93	
No	32	21		32	21	

Table S2
Clinicopathological characteristics according to MMP-9 QIF scores in CD68 and CD163 compartments in cohort A

COHORT A	MMP-9 Low in CD68+/CD163+	MMP-9 High in CD68+/CD163+	p-value	MMP-9 Low in CD68+	MMP-9 High in CD68+	p-value
**ER**						
**ER Negative**	17	36	**0.02**	18	35	**0.02**
**ER Positive**	47	44		48	43	
**PR**						0.07
**PR Negative**	18	37	**0.03**	20	35	
**PR Positive**	37	42		44	41	
**HER2**						0.5
**HER2 Negative**	22	24		23	23	
**HER2 Positive**	2	6	0.21	3	5	
**Size**			0.83			0.25
**<2cm**	42	49		46	45	
**>2 cm**	28	25		26	37	
**Age**			0.17			0.14
**<50 Years**	24	34		24	34	
**50 or >50 years**	58	58		59	50	
**LN Status**			0.8			0.8
**LN neg**	60	72		61	71	
**LN pos**	5	7		6	6	
**Grade**			0.24			0.18
**1or 2**	17	32		19	30	
**3**	11	35		12	34	
**Recurrence**			0.22			0.44
**Yes**	24	18		23	19	
**No**	59	68		61	66	

Table S3
Clinicopathological characteristics according to CD68 and CD163 QIF scores in cohort B

COHORT B	CD68 Low	CD68 High	p-value	CD163 Low	CD163 High	p-value
**Size**			0.18			0.53
<2cm	15	10		12	11	
>2 cm	20	26		19	24	
**Age**			0.12			0.5
<50 Years	14	25		17	19	
50 or >50 years	27	20		20	23	
**LN Status**			0.25			0.51
LN neg	11	4		9	4	
LN pos	8	7		8	6	
**Grade**			0.94			0.31
1or 2	11	12		11	9	
3	23	26		20	28	
**Stage**			0.38			0.6
I-II	14	15		14	2	
III-IV	1	3		12	1	

Table S4
Clinicopathological characteristics according to MMP-9 QIF scores in CD68 and CD163 compartments in cohort B

COHORT B	MMP-9 Low in CD68+/CD163+	MMP-9 High in CD68+/CD163+	p-value	MMP-9 Low in CD68+	MMP-9 High in CD68+	p-value
**Size**			0.06			0.13
<2cm	14	7		13	8	
>2 cm	18	25		18	25	
**Age**			0.24			0.25
<50 Years	15	19		15	19	
50 or >50 years	21	21		20	22	
**LN Status**			0.42			0.45
LN neg	10	7		11	5	
LN pos	4	6		6	5	
**Grade**			**0.01**			**0.009**
1or 2	13	5		13	5	
3	18	29		17	30	
**Stage**			0.76			0.7
I-II	15	2		15	10	
III-IV	11	1		2	2	
